# Perforation of the Urinary Bladder Caused by Transurethral Insertion of a Pencil for the Purpose of Masturbation in a 29-Year-Old Female

**DOI:** 10.1155/2010/460385

**Published:** 2010-08-25

**Authors:** Athanasios Bantis, Petros Sountoulides, Christos Kalaitzis, Stelios Giannakopoulos, Eleni Agelonidou, Soultana Foutzitzi, Stavros Touloupidis

**Affiliations:** ^1^Department of Urology, University Hospital of Alexandroupolis, Dragana, 68100 Alexandroupolis, Greece; ^2^Department of Radiology, University Hospital of Alexandroupolis, Dragana, 68100 Alexandroupolis, Greece

## Abstract

The urethra is a usual site of introduction of foreign bodies for autoerotic stimulation. We present an unusual case of bladder perforation caused by foreign body that was self-inserted in the urethra and consequently slipped inside the bladder in a 29-year-old female patient with psychiatric disease. The patient was referred to our department for macroscopic hematuria and abdominal pain. Imaging studies revealed the presence of a foreign body in the pelvic area which had perforated the left lateral wall of the bladder. The foreign body was removed via open cystotomy. In psychiatric patients hematuria and pelvic pain may result from insertion of a foreign body in the bladder usually during masturbation.

## 1. Introduction

Foreign Bodies (FBs) usually enter the bladder via migration from the urethra either during clitoris or penis masturbation. Other rare causes are iatrogenic or migration from surrounding structures, vagina, cervix, uterus, or rectum. The aim of our study is to discuss the diagnostic and therapeutic implications in a challenging case of bladder perforation caused by the insertion of a foreign body in the bladder during masturbation.

## 2. Case Presentation

**Figure 1:**
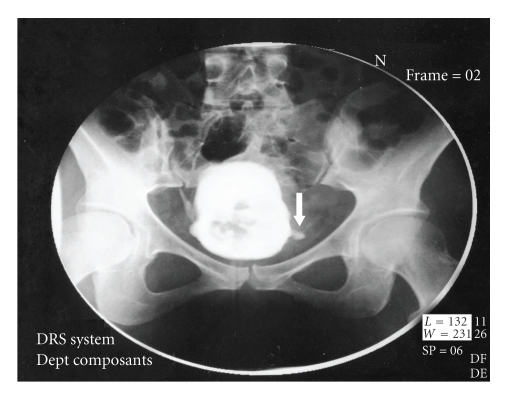
X-ray of bladder: the foreign body penetrate the left wall of bladder.

**Figure 2:**
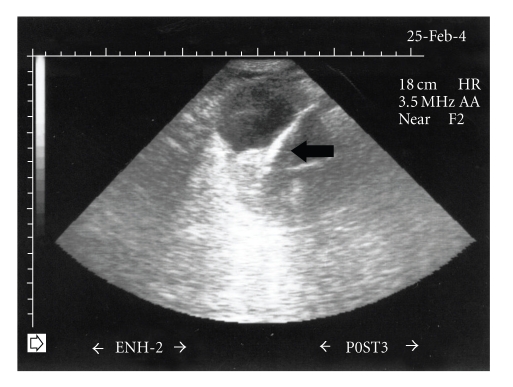
Ultrasonography shows a straight echogenic foreign body (arrow).

**Figure 3:**
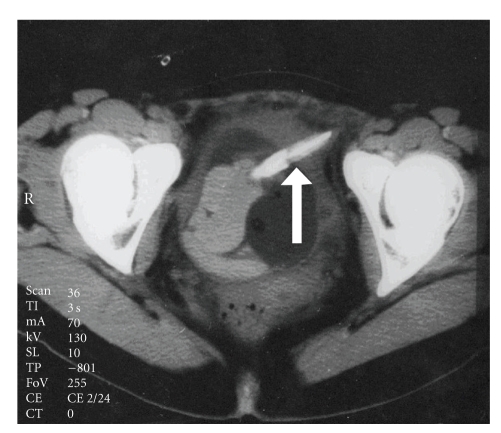
CT scan through the mid pelvis demonstrates a foreign body in urinary bladder which penetrates the right side (arrow).

**Figure 4:**
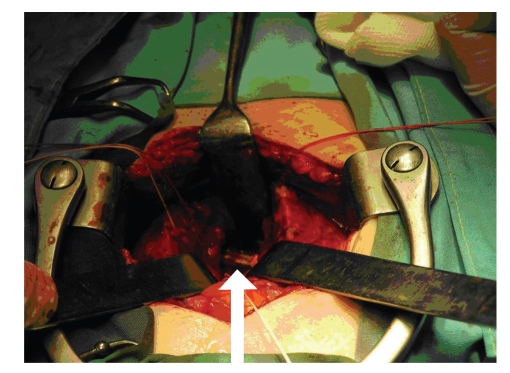
Intraoperative view through a suprapubic cystotomy (arrow).

**Figure 5:**
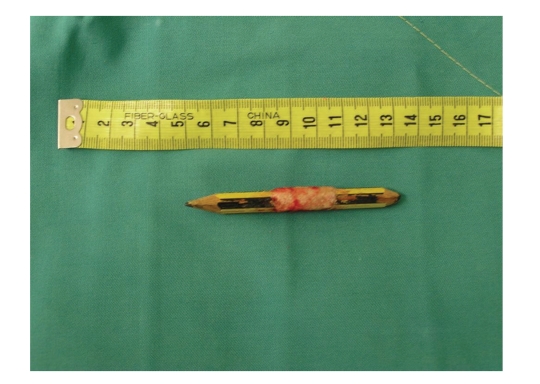
The piece of pencil after the surgical extraction.

We present the case of a 29-year-old female suffering from dementia which was eventually found to have inserted a pencil into the urethra for the purpose of masturbation. The pencil accidentally slipped into the urinary bladder and the patient was referred to the emergency room a few days after the incident due to macroscopic hematuria, dysuria, and lower abdominal pain. On initial questioning the patient did not reveal the insertion of FB in her urethra. Therefore, imaging studies were undertaken as in the routine evaluation of macroscopic hematuria. Bladder ultrasonography (US) (Figure 1), and intravenous urography (IVU) confirmed the presence of an object in the shape of a small pencil inside the bladder. IVU documented the clinical suspicion of bladder perforation caused by a sharp piece of wood penetrating the left lateral bladder wall. Perforation was confirmed by the escape of contrast media outside the bladder at the left bladder wall (Figure 2). Abdominal CT scan was performed to confirm the presence of a foreign body penetrating the left lateral wall of the urinary bladder and exclude concomitant trauma of adjacent organs (Figure 3). We decided against the endoscopic removal of the FB due to the large diameter of the pencil and the risk of additional injury to the bladder and urethra with the use of graspers. Therefore, the patient underwent open cystotomy under general anesthesia for removal of the piece of pencil (Figures 4 and 5). The bladder was closed in two layers and a 20 Ch Foley catheter and a small perivesical drain were left. The patient had an uneventful recovery and was discharged home after 3 days. The catheter was removed on the seventh postoperative day.

## 3. Discussion

A wide range of foreign bodies that were self-introduced in the urethra and bladder has been reported in both sexes. The insertion of objects such as eyebrow pencil, wrist watch, Blu-Tack, cable, rubber tube, electrical wire, cocaine, hair, ballpoint pen, or even cucumber has been reported in the literature [1–9]. 

The most common reason for self-insertion of a foreign body into the urethra is of erotic or sexual nature, usually for masturbation [10–13]. The majority of cases are associated with dementia, other psychiatric abnormalities, or drug intoxication [1, 3, 4, 6]. The iatrogenic introduction of foreign bodies has also been reported. Iatrogenically introduced foreign bodies include retained urethral catheter tip, tip of ureteric catheter, broken stent, beak of cystoscope, filiform guide, thermometer tip, retained gauze piece, retained inflated balloon of a Foley catheter, and suture material [7, 14]. 

In the majority of these cases clinical presentation varies from asymptomatic to swelling of the external genitalia, dysuria, poor urinary stream or retention, bloody or purulent urethral discharge, and ascending urinary tract infection [10, 11]. In our case the configuration and nature of the foreign body led to perforation of the bladder with signs and symptoms of acute abdomen. In a case similar to our case of bladder perforation a ballpoint pen that was inserted in the bladder was discovered years later in the retroperitoneal space [9]. 

With regard to management and depending on the type of foreign body and its location, various methods of removal have been described, including meatotomy, cystoscopy, internal or external urethrotomy, suprapubic cystostomy, Fogarty catheterization, and injection of solvents [15]. Endoscopic therapy is usually the first line option. Although in the majority of cases foreign bodies can be relatively easily removed, in some cases there might be significant difficulty in removal of a foreign body requiring the use of unusual equipments such as Amplatz dilators or even nephroscopes for the retrieval of screws as well as magnetic retrievers for galvanic objects [10]. However, in the majority of mobile objects inside the urethra, it is relatively easy to push the objects towards the bladder where the foreign body can be grasped with forceps or retrieval baskets. The YAG laser has lately been reported to have been used for this purpose [2]. 

In cases where endoscopic techniques are unsuccessful, then one has to resolve to open surgery as in our case. Endoscopic retrieval of a foreign body from the male urethra and bladder may be more challenging due to the longer urethral length and the occasional presence of obstructive BPH in older men. On the contrary, endoscopic retrieval is facilitated by the shorter length and wider diameter of the female urethra. For objects stuck in the penile urethra, external urethrotomy is recommended [16], while for intravesically located foreign bodies, a suprapubic cystotomy is necessary for removal of the foreign body under direct vision [8].

## 4. Conclusions

The case presents a potentially serious complication of an accidentally introduced foreign body into the urinary bladder during masturbation. Macroscopic hematuria and abdominal pain should raise the suspicion of a foreign body insertion in the urinary bladder, especially in psychiatric patients.
